# The Beneficial Role of Auricular Point Pressure in Insomnia and Anxiety in Isolated COVID-19 Patients

**DOI:** 10.1155/2021/6611942

**Published:** 2021-07-08

**Authors:** Yueming Luo, Chuanren Ling, Yangchen Liu, Chong Deng, Ana Maria Waaga-Gasser, Minggui Chen, Zehui He, Erhui Chen, Lin Wei, Shimiao Luo, Xiaozhen Gong, Hong Ye, Jing Zhu, Shan Song, Qiuting Wang, Shunmin Li, Martin Gasser, Meizhen Lin

**Affiliations:** ^1^The Fourth Clinical Medical College of Guangzhou University of Chinese Medicine, Shenzhen Traditional Chinese Medicine Hospital, Shenzhen, China; ^2^The Second Affiliated Hospital of Guangzhou University of Chinese Medicine, Guangzhou, China; ^3^Guangzhou University of Traditional Chinese Medicine, Guangzhou, China; ^4^Brigham and Women's Hospital, Harvard Medical School, Boston, MA, USA; ^5^Hunan University of Traditional Chinese Medicine, Changsha, China; ^6^University of Wuerzburg, Wuerzburg, Germany

## Abstract

**Background:**

Coronavirus disease 2019 (COVID-19) causes psychological distress and can have a negative impact on the general mental health and rehabilitation in affected patients under currently implemented isolation guidelines. Auricular point pressure (APP) as well-established technique in traditional Chinese medicine may help to relieve sleep disturbance and anxiety in COVID-19 patients.

**Methods:**

During the early phase of the epidemic/pandemic, patients were enrolled in this study (02/2020 until 03/2020 *n* = 84). They were strictly isolated on specific wards at the Hubei Provincial Hospital of Integrated Chinese and Western Medicine in Hubei. The retrospective cohort study design included two groups. Group A patients were treated with an auricular point pressure (APP) in addition to standard intensive care medicine while Group B participants (No-APP) received routine nursing measures alone. Treatment outcome was measured using the St. Mary's Hospital Sleep Questionnaire (SMH) Score and the 7-Item Generalized Anxiety Disorder Scale (GAD-7). Both scores were measured in each patient at baseline and on the discharge day.

**Results:**

The SMH score and sleep status changed in APP patients at the end of the treatment period when compared with No-APP patients (*P* < 0.01). APP-treated patients demonstrated lower GAD-7 scores than No-APP controls (*P* < 0.01). Further, no significant differences in safety or adverse events between the APP and No-APP groups were observed.

**Conclusion:**

The results from our snapshot study during the early phase of the SARS-CoV-2 epidemic/pandemic suggest that auricular point pressure could be a simple and effective tool to relieve insomnia and situational anxiety in hospitalized patients suffering from COVID-19 and kept under disconcerting conditions of isolation.

## 1. Introduction

COVID-19 is an infectious disease caused by the severe acute respiratory syndrome coronavirus 2 (SARS-CoV-2). It spread out rapidly worldwide which started in late December 2019 [1]. According to data released by John's Hopkins University in Baltimore, USA, the number of patients with confirmed COVID-19 worldwide has meanwhile increased to more than 152 million as of May 3, 2021, and more than 3 million of affected patients worldwide have died. Unfortunately, there are still no proven specific therapies for COVID-19 to prevent SARS-CoV-2 infection, and experiences from previous observations during the coronavirus epidemic in 2002/2003 have shown similarities with current difficulties in establishing effective treatment strategies for COVID-19 [2, 3]. As a result, the SARS-CoV-2 pandemic has substantially increased the risk of death from infection and thereby also results in severe psychological pressure for the isolated patients [4, 5].

Rigorous isolation with direct contact only to almost not identifiable medical and nursing staff under personal protective equipment (PPE) may trigger additional psychological distress in COVID-19 patients suffering from respiratory and other serious health problems. Therefore, recovery from physical and psychological limitations seems to be negatively influenced through needed quarantining and contact restrictions during the acute phase of COVID-19 as well as the following rehabilitation phase [6, 7]. The isolation with specific hygiene requirements on intensive care units and following social isolation and economic uncertainty have led to a considerable increase in consecutive mental health concerns including loneliness, anxiety, depression, and suicidal ideation [8]. To avoid losses caused by such crisis events, patient guidance regarding effective and appropriate strategies to regulate emotional burden has become a priority.

Traditional Chinese medicine (TCM) has been used for more than 2,000 years and plays an important role alongside of contemporary medicine in China [9]. TCM has demonstrated effectiveness in the management of coronavirus infection [10, 11]. Auricular point pressure (APP) is a well-known component of TCM therapy that refers to acupoints distributed on the auricle. The method involves placement of a small seed (*Vaccaria segetalis*) over the appropriate acupressure point on the ears; the seed is held in place with an adhesive material, thereby stimulating the meridians. This influences the release of neurotransmitters from neurons and regulates functions of the endocrine system and viscera. As a result, it can reduce symptoms of discomfort and can help to successfully treat several diseases [12].

Despite the benefits of APP, no clinical trials have been documented so far to evaluate its effects in patients with COVID-19. Therefore, this study was performed to investigate the safety and efficacy of APP in patients with COVID-19.

## 2. Method

### 2.1. Study Design and Participants

A retrospective cohort study was conducted at the Hubei Provincial Hospital of Integrated Chinese and Western Medicine in Hubei Province with a total of 84 patients with COVID-19 (aged between 30 and 91 years, mean age 59.4 ± 15.4) that participated in this study after confirmed consent (02/2020 until 03/2020). Eligible patients were assigned to either the APP group (Group A: APP plus regular nursing) or the control group (Group B: regular nursing alone) on their own will. Patients in Group A received APP in accordance with evidence-based principles. The patients and researchers were not blinded to treatment allocation.

Prior to the procedure, the TCM nurses thoroughly washed their hands and disinfected each acupressure and then dried. *Vaccaria segetalis* seeds were placed with a piece of tape on the following ear acupoints until the patient felt aching, numbness, distention, or pain: Fei (CO14), Jiaogan (AH6a), Shenmen (TF4), Pizhixia (AT4), and Neifenmi (TF2). They remained attached on the ear and were pressed by the patient for one minute at 4- to 6-hour intervals. Patients were also instructed to press the point 15–30 min before bedtime. The seeds were replaced and added to the other ear at 3-day intervals.

The main endpoints investigated were symptoms of depression, anxiety, insomnia, and distress for all participants. The 7-Item Generalized Anxiety Disorder (GAD-7) scale (range, 0–21) and the St. Mary's Hospital (SMH) Sleep Questionnaire were used to assess the severity of anxiety and sleep symptoms, respectively. The endpoints were measured using the Chinese versions of these validated measurement tools.

### 2.2. Ethics Statement

The study was undertaken in compliance with the principles of the Declaration of Helsinki. Ethics approval was reviewed and approved by the hospital's Human Research Ethics Committee (no. ZE2020-032-01). The clinical trial has been publicly registered in the National Health Insurance Information Platform for Medical Research Registration and Recording Information System (no. MR-44-20-002619) on March 14, 2020.

The trial data and statistical analysis were handled by the author to maintain patient confidentiality; collected data were stored in a locker in the private office of the author.

### 2.3. Patient Characteristics

The participants were chosen based on their clinical characteristics and the presence of laboratory-confirmed COVID-19, based on the “Pneumonia Diagnosis and Treatment Protocol for Novel Coronavirus (SARS-CoV-2) Infected Pneumonia” (trial version 5) [11]. Prior to any intervention, all participants were assessed for disease-related characteristics, sleep score, sleep satisfaction score, and anxiety level.

The inclusion criteria were as follows: (1) diagnosis of pneumonia based on clinical manifestations and abnormal findings in chest X-rays or computed tomography scans and (2) confirmation of COVID-19 by high-throughput sequencing or real-time reverse transcriptase polymerase chain reaction assays from nasal and pharyngeal swab specimens [13]. The exclusion criteria were as follows: (1) presence of severe heart, liver, brain, kidney, and/or other severe physical and mental diseases; (2) presence of language and/or cognitive impairment; and (3) ongoing treatment with antianxiety or anti-insomnia medications.

### 2.4. Preparation of *Vaccaria segetalis* Formulation

The *Vaccaria segetalis* seed was purchased from Shanghai Taicheng Tech & Development Co., Ltd. (Shanghai, China; batch no. 20160002).

### 2.5. Baseline and Outcome Assessment

During enrollment, participants' demographic data were collected regarding sex, age, and dependence on assistance from an attendant. Study outcome measures were assessed from baseline until the day of discharge (i.e., end of intervention therapy) (Table 1).

The primary outcome parameters were sleep condition events associated with SMH score from baseline to follow-up. The SMH is a systematic inquiry into a patient's sleep experience and comprises items of demonstrable reliability [14, 15]. The SMH scores obtained at baseline and on the last day of the study were compared between groups.

The prespecified secondary outcome was the GAD-7 score. The GAD-7 is a one-dimensional instrument designed to detect symptoms of generalized anxiety disorder [16]. The GAD-7 includes seven items based on seven core symptoms and assesses the frequencies with which respondents experience these symptoms within the prior 2 weeks [16]. Respondents report their symptoms using a 4-item Likert rating scale ranging from 0 (not at all) to 3 (almost every day); the total score ranges from 0 to 21 [16]. The GAD-7 is a well-validated screening instrument and has demonstrated excellent internal consistency (Cronbach's *ɑ* = 0.911). GAD-7 descriptors were translated into Chinese for this study.

### 2.6. Trial Assessments

**Figure 1:**
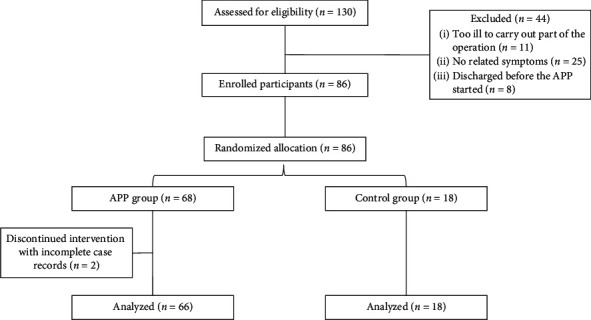
Flowchart of the clinical study.

During the trial, patients in both groups were visited at baseline and on the day of discharge following the first treatment. All patients completed the SMH and GAD-7 assessment (Figure 1).

### 2.7. Statistical Analysis

In this study, continuous variables were expressed as means and standard deviations or standard errors of the mean, as specified. They were analyzed together with summarized categorical data as frequencies or percentages. The statistical analyses were performed using IBM SPSS Statistics, version 22.0 (IBM Corp., Armonk, NY, USA). The general descriptive characteristics were calculated, including baseline patient characteristics and outcomes. The primary outcome was based on the GAD-7 for anxiety. Secondary endpoints were the SMH for the patients' sleep conditions. The *χ*^2^ test or Fisher's exact test was used to compare percentages between groups. Data were carefully reviewed and confirmed by an experienced physician team to ensure their accuracy.

### 2.8. Adverse Event (AE) Safety Monitoring

In this study, the safety profiles of the study interventions in both groups were assessed by the number of AEs reported during the treatment period. When an AE occurred, the investigator evaluated the outcome and its relevance to the study. Based on its relevance, a decision was made regarding continued observation.

## 3. Results

### 3.1. Participant Characteristics

Eighty-four patients with confirmed COVID-19 who were admitted to the Hubei Provincial Hospital of Integrated Chinese and Western Medicine in Hubei were included in the final analysis. Recruited patients were assigned to the APP group (Group A: *n* = 66) or control group (Group B: *n* = 18) (Figure 1). Baseline characteristics were comparable between both groups (Table 1). The mean patient ages were 59.0 ± 15.5 years in the APP group and 60.8 ± 15.7 years in the control group. The treatment days were 11.32 ± 8.2 in the APP group and 8.7 ± 4.8 in the No-APP group. There were no statistically significant differences between the two groups in demographic data or baseline GAD-7 and SMH scores. In the baseline geographic characteristics, both the GAD-7 score and SMH score did not significantly differ between the two groups (*P* > 0.05, Tables 2 and 3).

### 3.2. Effect of APP on Anxiety Outcomes

**Figure 2:**
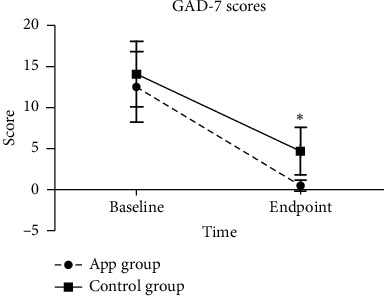
Comparison of the generalized anxiety disorder scale (GAD-7) between the auricular point pressure (APP) group and the No-APP group. ^*∗*^Significant difference compared with the control group, *P* < 0.05.

Both groups showed significantly lower GAD-7 scores compared to baseline while intergroup comparison demonstrated for the APP group patients a significantly lower GAD-7 score (0.5 ± 0.7) compared with the control group (4.7 ± 2.9; *P* < 0.05) (Figure 2). Analysis of the subgroup scores by rank sum test showed for all APP-treated patients at their day of discharge (*n* = 38) a score lower than 4, while in the No-APP group eight patients still demonstrated scores between 5 and 9 and one patient a score higher than 10. This trend clearly showed for the APP group a better GAD-7 outcome score (Table 4). Moreover, whenever the patients received the treatment (either at the beginning of their COVID-19 disease course or a week after becoming isolated and treated over 14 days), the intervention with APP effectively alleviated anxiety in patients with COVID-19 (Table 2).

### 3.3. Effect of APP on Sleep Outcome

**Figure 3:**
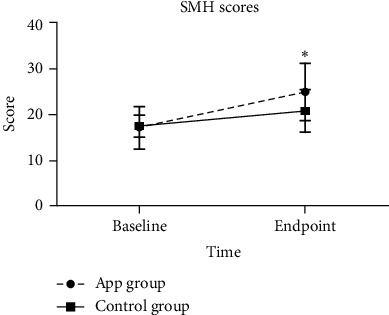
Comparison of the St. Mary's Hospital Sleep Questionnaire (SMH) scores between the APP group and the No-APP group. ^*∗*^Significant difference compared with the control group, *P* < 0.05.

An independent sample test was used to compare the SMH scores between groups. Patients in the APP group had a significantly higher SMH score (*P* < 0.01) than patients in the control group at their respective study endpoints (Figure 3); this suggested that patients in the APP group showed a better sleep quality following treatment than patients in the control group. In addition, patients who received APP intervention at the beginning of the disease (*n* ≤ 7) and when the disease lasted over 14 days demonstrated lower SMH scores (*P* < 0.05) (Table 3).

### 3.4. Adverse Events

No serious adverse events in the treatment group and no significant differences in safety or adverse events (AE) between the APP and the control group were observed during the study. Most patients tolerated APP well.

## 4. Discussion

The COVID-19 pandemic has profoundly affected the health and livelihood of millions of people worldwide at an unprecedented scale and speed. Fortunately, vaccination programs have most recently been started in many countries after tremendous efforts in vaccine development within the last 12 months since the outbreak in 2020. This gives hope and encourages people to become actively immunized with one of the meanwhile available vaccines against SARS-CoV-2 for antibody-based protection against severe courses of COVID-19 [17]. However, in many countries infection rates remain at high numbers and much more corona vaccines are still needed. Importantly, since the beginning of the pandemic, infected patients with COVID-19 have been strictly isolated. As a result, many COVID-19 patients feel socially isolated, perceive danger, uncertainty, and report of significant physical discomfort. They fear of virus transmission to others and are confronted with overwhelming negative news portrayal in mass media coverage. Patients suffering from COVID-19 under such circumstances may experience loneliness, anger, anxiety, depression, and insomnia as well as posttraumatic/postinfectious stress symptoms [18]. Accordingly, the pervasive stress and social/environmental disruptions from the COVID-19 pandemic have also impacted sleep in affected patients. A number of reports suggest that COVID-19 is likely to have a significant impact on psychological distress [19, 20]. Anxiety disorders have been linked to sleep disturbance in affected individuals. Patients with COVID-19 in worse condition typically exhibit increased anxiety and sleep disturbance. Furthermore, patients with higher levels of anxiety and sleep disturbance showed worse outcome [21, 22]. Therefore, methods to alleviate patient anxiety are necessary. Given that TCM is an important part of contemporary medicine in China, its clinical effectiveness in the treatment of COVID-19 has been gaining increasing attention [23].

Auricular points refer to acupoints distributed on the auricle, and auricular point pressure (APP) represents a subdiscipline of both acupuncture and TCM. APP can stimulate the meridians because it applies pressure to an acupoint. Each of the acupoints reflect the physiological and pathological condition of its corresponding body part and must be stimulated by pressure or by a needle to regulate the dysfunction in that body part [12]. Specifically, the effect is triggered by pressure on a specific acupoint and is accompanied by a warm, swelling pain sensation (qi sensation).

However, the desired effect is not simply elicited by manual pressure. APP consists of the application of pressure to specific locations on the ear. The intervention by auricular acupressure involves the placement of a *Vaccaria segetalis* seed (with an adhesive material) over the appropriate acupressure point on the ears. Wangbuliuxing, the seed of *Vaccaria segetalis*, is native to temperate zones in Asia, Africa, and Europe. It has been used for the treatment of amenorrhea, mastitis, and urinary diseases in TCM [24]. The main active components include the triterpenoid saponins, cyclic peptides, and flavonoids [25]. Additionally, pressure on the auricular acupoint has been shown to ease anxiety in several studies [12].

In addition, APP can also be achieved manually (i.e., acupressure) with an acupuncture needle, laser, or a magnet. All of this can be used as complementary treatment and generally contribute to improvement of symptoms and reduction of exacerbation. It influences the release of neurotransmitters which transmit signals along neurons, thereby regulating the function of endocrine organs and the viscera. These neuroregulatory changes can reduce symptoms of discomfort and cure several diseases [12, 26].

Previous studies have shown that this alternative, a nonpharmacological APP treatment, can relieve a variety of distressing symptoms including dyspnea [27], amenorrhea, edema [28], and fatigue [12]. Application of pressure to specific locations on the auricle can also improve postpartum anxiety [12] and sleep quality [29]. Notably, the biological basis for sleep disorders is linked to arousal of the central or autonomic nervous system; stimulation of acupressure points can stabilize the autonomic and central nervous system by directly affecting peripheral nerves and muscles [30]. Therefore, the improvement in sleep quality observed in our study may have been elicited by a more balanced sympathetic/parasympathetic nervous system activity, thus less sympathetic and comparably more parasympathetic nervous system signaling. Notably, the same result was found in a systematic review regarding the effect of APP in patients with insomnia [31].

Our findings from a pilot cohort study have shown that auricular acupressure could reduce anxiety and improve sleep quality in patients with COVID-19. The results are consistent with data from previous research [32, 33] and also reports which demonstrated that APP effectively reduced anxiety symptoms on the background of different etiologies (e.g., perioperative anxiety, caesarean section [12], and healthcare provider situational stress and anxiety [34]). Particularly, the improvement of anxiety levels during strict isolation of infectious COVID-19 patients during the first wave of the pandemic may have resulted in positive effects on the overall recovery process from this devastating disease and would support reports from others on the impact of stress through patient isolation [6, 7]. However, this remains so far speculative as it was not specifically investigated in our cohort.

The design process was rigorous and the trained medical staff involved in the research strictly followed the standard operating procedures. However, this pilot study also had several limitations. First, it was limited in sample size; given the COVID-19 pandemic, a blinded or sham control group could not be included. Second, the study was carried out within a short treatment period of 10 days, with assessment only on two occasions at the beginning and at the end of the treatment interval, and almost equally important, the study lacked a longitudinal follow-up. More precisely, the long-term psychological changes of patients in this population are worth of further investigation. Third, with SMH and GAD-7 scores, it is only to a limited extent possible to provide a reasonably accurate assessment of the severity and impact of side effects experienced by patients with COVID-19. Nevertheless, our data represent a snapshot that demands for larger randomized studies with more longitudinal assessment of the study population to reinforce our findings.

In conclusion, our results suggest that auricular point pressure could be a promising additional tool in the multimodal treatment for improving outcome in patients with COVID-19. Patients in the APP treatment group showed considerable symptom relief (sleep disturbances and anxiety) and improved quality of life, as indicated by the SMH and GAD-7 scores. Moreover, our results demonstrated APP as a safe and easily applicable treatment in patients with COVID-19. Further clinical studies are necessary to more comprehensively evaluate the application of APP in these patients over longer time periods.

## Figures and Tables

**Table 1:** Baseline demographics.

Characteristic	Group A APP^*∗*^ (*n* = 66)	Group B No-APP (*n* = 18)	*P*
Age (years)	59.0 ± 15.5	60.8 ± 15.7	0.658
Sex			
Male (%)	33 (50.0)	6 (33.3)	0.209
Female (%)	33 (50.0)	12 (66.6)	
Observation time (days)	11.32 ± 8.2	8.7 ± 4.8	0.089

^*∗*^APP: auricular point pressure.

**Table 2:** Generalized anxiety disorder scale (GAD-7) scores before and after intervention by intervention time.

Intervention time (days)		Group A APP^*∗*^ *N*, mean (SD)	Group B No-APP *N*, mean (SD)	*Z*	*P*
At the beginning of the disease (*n* ≤ 7)	Day 0	14, 12.6 (3.9)	3, 15.0 (4.6)	−0.950	0.342
	Day of discharge	5, 0.2 (0.4)	3, 4.7 (2.3)	−2.399	0.016

After a week when they are diagnosed (7 ≤ *n* ≤ 14)	Day 0	13, 15.4 (2.8)	2, 17.0 (2.8)	−0.688	0.491
	Day of discharge	10, 0.3 (0.7)	2, 4.5 (2.1)	−2.556	0.011

The disease lasted over 14 days	Day 0	36, 11.4 (4.4)	13, 13.5 (4.1)	−1.187	0.235
	Discharge	23, 0.6 (0.7)	13, 4.7 (3.2)	−4.355	<0.001

Total	Day 0	63, 12.5 (4.3)	18, 14.1 (4.0)	−1.229	0.219
	Discharge	38, 0.5 (0.7)	18, 4.7 (2.9)	−5.691	<0.001

^*∗*^APP: auricular point pressure.

**Table 3:** St. Mary's Hospital Sleep Questionnaire (SMH) scores before and after intervention.

Intervention period (days)		APP^*∗*^ group *N*, mean (SD)	Control group *N*, mean (SD)	Z	*P*
Treatment starting at the beginning of the disease and isolation period (days of treatment ≤ 7)	Day 0	15, 16.2 (7.2)	3, 16.7 (2.5)	−0.420	0.738
	Discharge	14, 23.1 (7.4)	3, 19.0 (1.0)	−2.278	0.023

Treatment starting one week after the diagnosis and isolation (days of treatment > 7 ≤ 14)	Day 0	13, 16.4 (2.9)	2, 19.5 (0.7)	−1.720	0.085
	Discharge	13, 25.8 (5.2)	2, 17.5 (3.5)	−1.877	0.061

Disease and treatment lasting >14 days	Day 0	38, 17.5 (3.8)	13, 17.2 (2.5)	−0.544	0.587
	Discharge	37, 25.1 (6.1)	13, 21.6 (5.1)	−2.198	0.028

Total	Day 0	66, 17.0 (4.6)	18, 17.4 (2.4)	−0.071	0.943
	Discharge	64, 24.8 (6.2)	18, 20.7 (4.6)	−3.313	0.001

^*∗*^APP: auricular point pressure.

**Table 4:** Generalized anxiety disorder scale (GAD-7) scores before and after intervention by different score.

		GAD-7 score	Group A APP^*∗*^	Group B No-APP	*Z*	*P*
Anxiety	Day 0 (*N* = 81)	0–4	4	0	−1.124	0.261
		5–9	11	4		
		10–14	21	3		
		≥15	27	11		
	Day of discharge (*N* = 56)	0–4	38	9	−4.708	<0.001
		5–9	0	8		
		10–14	0	1		
		≥15	0	0		

^*∗*^APP: auricular point pressure.

## Data Availability

The datasets used and/or analyzed during the current study are available from the corresponding author upon reasonable request.
